# Estimating the Lineage Dynamics of Human Influenza B Viruses

**DOI:** 10.1371/journal.pone.0166107

**Published:** 2016-11-09

**Authors:** Mayumbo Nyirenda, Ryosuke Omori, Heidi L. Tessmer, Hiroki Arimura, Kimihito Ito

**Affiliations:** 1 Graduate School of Information Science and Technology, Hokkaido University, Sapporo, Hokkaido, Japan; 2 Division of Bioinformatics, Research Center for Zoonosis Control, Hokkaido University, Sapporo, Hokkaido, Japan; 3 JST, PRESTO, 4-1-8 Honcho, Kawaguchi, Saitama, 332–0012, Japan; Korea University College of Medicine and School of Medicine, REPUBLIC OF KOREA

## Abstract

The prediction of the lineage dynamics of influenza B viruses for the next season is one of the largest obstacles for constructing an appropriate influenza trivalent vaccine. Seasonal fluctuation of transmissibility and epidemiological interference between the two major influenza B lineages make the lineage dynamics complicated. Here we construct a parsimonious model describing the lineage dynamics while taking into account seasonal fluctuation of transmissibility and epidemiological interference. Using this model we estimated the epidemiological and evolutional parameters with the time-series data of the lineage specific isolates in Japan from the 2010–2011 season to the 2014–2015 season. The basic reproduction number is similar between Victoria and Yamagata, with a minimum value during one year as 0.82 (95% highest posterior density (HPD): 0.77–0.87) for the Yamagata and 0.83 (95% HPD: 0.74–0.92) for Victoria, the amplitude of seasonal variation of the basic reproduction number is 0.77 (95% HPD:0.66–0.87) for Yamagata and 1.05 (95% HPD: 0.89–1.02) for Victoria. The duration for which the acquired immunity is effective against infection by the Yamagata lineage is shorter than the acquired immunity for Victoria, 424.1days (95% HPD:317.4–561.5days). The reduction rate of susceptibility due to immune cross-reaction is 0.51 (95% HPD: 0.084–0.92) for the immunity obtained from the infection with Yamagata against the infection with Victoria and 0.62 (95% HPD: 0.42–0.80) for the immunity obtained from the infection with Victoria against the infection with Yamagata. Using estimated parameters, we predicted the dominant lineage in 2015–2016 season. The accuracy of this prediction is 68.8% if the emergence timings of the two lineages are known and 61.4% if the emergence timings are unknown. Estimated seasonal variation of the lineage specific reproduction number can narrow down the range of emergence timing, with an accuracy of 64.6% if the emergence times are assumed to be the time at which the estimated reproduction number exceeds one.

## Introduction

The influenza virus is one of the most common respiratory viruses and causes a high disease burden worldwide [1]. The influenza viruses co-circulating among humans can be classified as influenza A viruses (IAV) and influenza B viruses (IBV). Approximately 75% of confirmed cases of influenza are infections by the IAV [2]. IAV shows high antigenic diversity and rapid change in antigenicity, and appropriate intervention against IAV epidemics is quite difficult in terms of vaccine strain selection. The disease burden of IBV is also high, and 25% of confirmed cases of influenza virus infection and 22–44% of pediatric influenza related deaths in the US are caused by influenza B [2,3]. The number of major lineages of IBV is relatively low compared to type A. There are only two major genetically and antigenically distinct lineages; the Yamagata lineage and the Victoria lineage.

Trivalent vaccines against influenza include one of those two lineages. The selection of the correct vaccine lineage is essential for high vaccine efficacy against influenza B infections. Despite the limited number of existing IBV lineages, vaccine strain selection is still difficult because the dominant lineage changes over time and the switching time of the dominant lineage is difficult to predict. Although the quadrivalent vaccine includes both IBV lineages, Höpping et al. 2016 pointed out the necessity of vaccine strain selection because the use of trivalent vaccines is still common worldwide and the cost-effectiveness of quadrivalent vaccines is under debate [4].

**Fig 1:**
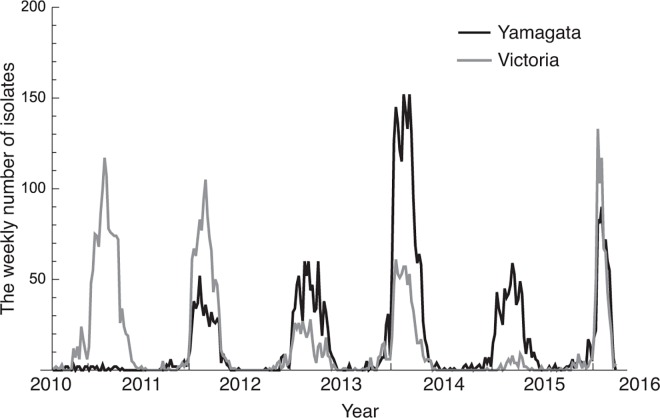
Time series data of weekly reported lineage-specific IBV cases isolated in Japan from the 2010–2011 season to the 2015–2016 season. The gray line shows the number of isolates of the Victoria lineage and the black line shows the number of isolates of the Yamagata lineage.

To predict an effective vaccine strain for IBV, a good model capturing the mechanisms of its complex dynamics is needed. Important factors to consider regarding the complex epidemic dynamics of the Yamagata and Victoria lineages are i) the seasonal variation of transmissibility, ii) epidemiological interference between the two lineages, and iii) time series changes of antigenicity due to the evolution of the pathogens. The incidence of IBV shows seasonal fluctuation which can be explained by the seasonality of the transmissibility of IBV. This transmissibility seasonality has been shown to be determined by seasonal variation of absolute humidity [5,6]. Previous theoretical studies have shown that seasonal variation in transmissibility can induce rich epidemic dynamics, such as periodic or chaotic behavior [7–9], therefore a model capturing seasonal fluctuation of transmissibility is essential to predicting epidemic dynamics. Epidemiological interference is also a known factor in complex epidemic dynamics [10–13]. The time series of confirmed cases between two lineages are negatively correlated (Fig 1), implying epidemiological interference between the two lineages. Moreover, vaccine efficacy studies also imply the existence of immune cross-reaction between lineages [4]. The epidemic dynamics of a lineage are affected by that of the other lineage assuming the existence of immune cross-reaction. The change of antigenicity of influenza across seasons is one major obstacle for prediction. Especially in the case of IAV, the large variety of lineages and epidemic interference between these lineages makes it difficult to predict the dynamics. To analyze these complex dynamics, models taking into account these complex evolutionary dynamics have been proposed so far [14–19]. Although the number of co-circulating IBV lineages is limited compared to IAV the large genetic diversity within the lineages and the changing antigenicity over time, especially for the Victoria lineage [20], makes dominant lineage prediction difficult. Modelling the complex evolutionary dynamics is essential to predicting future changes in IBV.

In this paper, we assess the predictability of the dominant lineage of IBV using a mathematical model describing IBV epidemics. To this end, we construct a parsimonious mathematical model that takes into account the seasonal variation of transmissibility, the epidemiological interference between lineages, and the time series change of antigenicity. We first estimate the parameters of our model, including these three factors, from the time series of IBV confirmed cases per lineage and time series of specific humidity. Using these estimated parameters we assess the predictive potential of the dominant lineage in the next season.

## Methods

### Data

We analyzed the weekly reports of the number of cases of human IBV in Japan from the 2010–2011 season to the 2015–2016 season, collected by the National Institute of Infectious Diseases, Japan (http://www.nih.go.jp/niid/en/influenza-e.html). The following analyses are based on the data which we accessed on 12th April 2016. Cases where the lineage was not available were excluded.

### Mathematical model describing natural history of IBV

We employed individual-based Monte Carlo simulation (IBM) with host populations of 10,000. The host population was determined based on a sensitivity analysis of posterior distributions to the host population size (S1 Fig). We described the transmission process of the Yamagata lineage and the Victoria lineage using the compartmental SEIRS model. Based on the natural history of IBV, we classified the host population into four classes by infection state against each lineage; susceptible *S*, latent *E*, infectious *I*, and recovered *R*. A total of 4^2^ = 16 infection states were considered. We denote these infection states by *XY* where *X* is the infection state against Victoria and *Y* is that for Yamagata. For example *SE* denotes *S* for Victoria and *E* for Yamagata.

**Fig 2:**
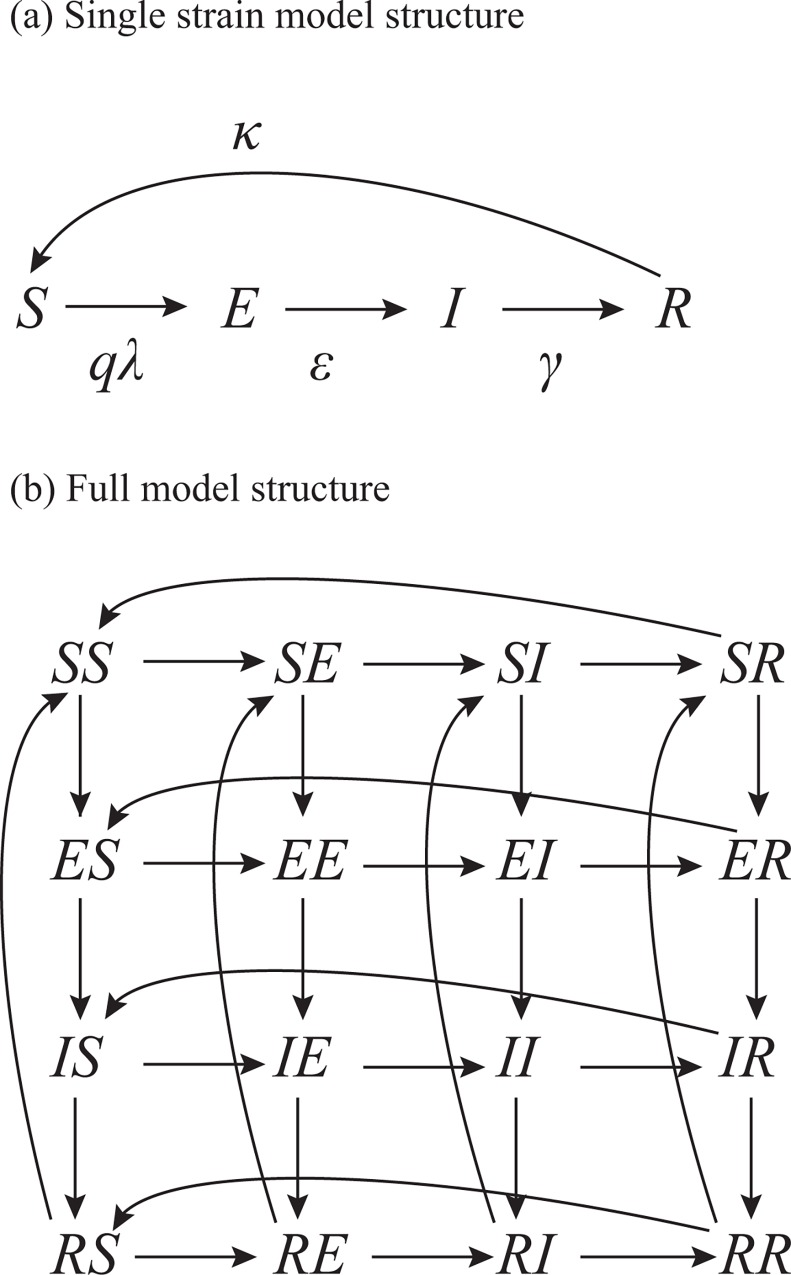
Model structure. (a) shows a single lineage model (one strain) and (b) shows the full model.

Fig 2A summarizes the transition of infection states for a lineage. The infection probability of a susceptible host is the product of the susceptibility of host *q* and the force of infection, *qλ*. Susceptibility of host *q* depends on infection states against both lineages as described below,

$$\begin{array}{l} q_{\text{victoria}}=\{\begin{array}{ll} 1 & \text{for}\;SS,SE,SI\\ \alpha_{\text{Yamagata}\to \text{Victoria}} & \text{for}\;SR\\ 0 & \text{otherwise} \end{array},\\ q_{\text{Yamagata}}=\{\begin{array}{ll} 1 & \text{for}\;SS,ES,IS\\ \alpha_{\text{Victoria}\to \text{Yamagata}} & \text{for}\;RS\\ 0 & \text{otherwise} \end{array}. \end{array}$$ (1)

For example, the susceptibility against Victoria is 1 if the host is susceptible to Victoria (the infection state for Victoria is *S*) and does not have any immunity against Yamagata (the infection state for Yamagata is *S* or *E* or *I*). If the host is susceptible to Victoria and has immunity against Yamagata (*SR*), the susceptibility to Victoria decreases to 1−*α*_Yamagata→Victoria_ by cross-immune reaction. The force of infection at time *t*, *λ*(*t*), is determined by the number of infected hosts *I*(*t*) and the specific humidity *h*(*t*) at time *t* as follows,

$$\begin{array}{l} \lambda_{n}(t)=\gamma_{n}\left[1+\exp\left(a_{n}-b_{n}h(t)\right)\right]\frac{I_{n}(t)}{N},\\ n\in \{\text{Victoria},\;\text{Yamagata}\}. \end{array}$$ (2)

Here *N* denotes the host population size, *γ* is the lineage specific recovery rate, the term 1+exp(*a*_n_-*b*_n_*h*(*t*)) describes the transmission coefficient determined by humidity, where *h* is specific humidity, and *a* and *b* are lineage specific parameters. We followed Shaman et al. 2010 [5] regarding the model of the relationship between specific humidity and transmissibility; i.e. specific humidity shows seasonal fluctuation resulting in seasonal fluctuation in transmissibility (and this fluctuation is reflected in parameter *b*). We extrapolated *h*(*t*) using the daily observed data of specific humidity in Tokyo, Japan, collected by the Japan Meteorological Agency (http://www.jma.go.jp/jma/indexe.html). *I*_n_(*t*) denotes the number of infected hosts with lineage *n*, for example, *I*_victoria_(*t*) = *IS*(*t*)+ *IE*(*t*)+ *II*(*t*) + *IR*(*t*). The host infection state becomes *E* after infection, and the host obtains infectiousness and the infection state becomes *I* with probability *ε*. *I* recovers with probability *γ* and obtains immunity. The immune response wanes due to the evolution of antigenicity. The emergence of new distinct lineages from existing lineages is not observed for a long time [20]. We assume that the evolutionary dynamics of IBV within the same lineage is stable and the probability of waning immunity is constant over time, *κ*. The parameters (*α*, *a*, *b*, *ε*, *γ*, and *κ*) are lineage specific.

### Estimation of parameters

Using the time-series data of the number of laboratory-confirmed IBV cases per lineage and specific humidity, we estimated the parameters (*α*, *a*, *b*, and *κ)* in the model described in the last section. Based on previous study [21] we parameterized *ε* as 1/*ε* = 0.6 day and *γ* as 1/*γ* = 4.0 day. These parameters are estimated for each lineage. We also estimated the herd immunity against each lineage at the beginning of the period explored in this study, i.e., *SS*, *SR*, *RS*, *RR* at the beginning of the 2010–2011 season. Hereafter, we refer to *SS*, *SR*, *RS*, *RR* at the beginning of the 2010–2011 season as *SS*(0), *SR*(0), *RS*(0), *RR*(0), respectively.

To estimate these parameters we implemented Approximate Bayesian Computation (ABC) using our model [22]. Models describing the interaction between nonlinear dynamics, i.e. epidemiological interference, are difficult to solve analytically. As a result, model-based inference is complicated to implement due to the difficulty of obtaining an analytical solution for the likelihood function. In such case ABC gives us a good approximation of the posterior distribution. The procedure of ABC that we conducted is, i) we simulated IBM with a parameter set, {*α*, *a*, *b*, *κ*, *SS*(0), *SR*(0), *RS*(0), *RR*(0), *p*}, determined by prior distribution of each parameter, ii) the simulation results were compared to the time-series data of lineage specific confirmed cases and we recorded the parameter sets, {*α*, *a*, *b*, *κ*, *SS*(0), *SR*(0), *RS*(0), *RR*(0), *p*}, if the distance between the simulation results and the observed data was smaller than a threshold, iii) we estimated prior distribution of each parameter (each element of parameter set) from the recorded parameter sets, {*α*, *a*, *b*, *κ*, *SS*(0), *SR*(0), *RS*(0), *RR*(0), *p*}, by kernel density estimation. To construct the posterior distributions of each parameter, we collected 1,000 parameter sets showing smaller distance between simulation and data than the threshold. The number of accepted IBM runs was determined based on sensitivity analysis of the number of accepted IBM runs to the posterior distributions (S2 Fig). We defined the distance between simulation results and observed data as D:

$$D\left(\alpha ,\;a,\;b,\kappa ,SS\left(0\right),SR\left(0\right),RS\left(0\right),RR\left(0\right),\;p\right)=\frac{p\sum_{t}\left|I_{sim}(t)-I_{obs}(t)\right|}{\sum_{t}I_{obs}(t)},$$ (3)

where *I*_sim_ denotes the simulation result of the number of infected individuals and *I*_obs_ denotes the field data. The parameter *p* is used for the adjustment of the population size. The parameter sets were accepted when the distance, *D*, is smaller than 0.44. We assume that the sampling probability of cases for laboratory testing and the host population size are constant over time and confirmed cases are proportional to the number of infected hosts. We set the prior distributions as uniform distributions for all parameters, the ranges of the priors are [0, 1] for *α*_Victoia→Yamagata_, [0, 1] for *α*_Victoia→Yamagata_, [0, 5] for *a*_Victoria_, [0, 5] for *a*_Yamagata_, [0, 5] for *b*_Victoria_, [0, 5] for *b*_Yamagata_, [0, 0.001] for *κ*_Victoria_, [0, 0.01] for *κ*_Yamagata_, [0, 10] for *p*, [0, 10000] for *SS*, [0, 10000] for *SR*, [0, 10000] for *RS*, [0, 10000] for *RR*. We normalized *SS*, *SR*, *RS*, and *RR* as *SS*+*SR*+*RS*+*RR* = 10000. We introduce the infected people at the beginning of each epidemic season as the initial condition during the IBM simulation process. We defined the beginning of the epidemic season as the time when the number of isolations exceeds 7. The number of infected people in the beginning of an epidemic season was adjusted by *p*.

### Prediction of dominant lineage

Using the posterior distributions obtained by ABC, we simulate IBM for one epidemic season and compare the results with empirical data of lineage specific confirmed cases. To measure the accuracy of the prediction, we conducted IBM 1,000 times and counted the number of simulations that showed the same dominant lineage as the empirical data. The average specific humidity at specific time points over the epidemic season was used for prediction. For prediction we consider three scenarios, i) predict using empirical data for the emergence dates of lineages, ii) predict without using empirical data for the emergence dates of any lineage, and iii) estimate the emergence dates of both lineages and predict the dominant lineage with the estimated emergence dates. In scenario ii), we simulated IBM while varying the emergence timing of both lineages from the beginning to the end of the epidemic season. Regarding iii), we assume the emergence timing for a lineage is equivalent to the time when the lineage specific basic reproduction number *R*_0,n_ exceeds one. In our model, *R*_0,n_ can be described by:

$$\begin{array}{l} R_{0,\;Victoria}(t)=\frac{SS+\alpha_{\text{Yamagata}\to \text{Victoria}}SR}{N}\\ \times \int_{\tau =t}^{\infty }\gamma \left[1+\exp\left(a_{Victoria}-b_{Victoria}h(\tau )\right)\right]\exp\left(-\gamma \tau \right)d\tau ,\\ R_{0,\;Yamagata}(t)=\frac{SS+\alpha_{\text{Victoria}\to \text{Yamagata}}RS}{N}\\ \times \int_{\tau =t}^{\infty }\gamma \left[1+\exp\left(a_{Yamagata}-b_{Yamagata}h(\tau )\right)\right]\exp\left(-\gamma \tau \right)d\tau . \end{array}$$ (4)

We estimate the timing when *R*_0,n_ exceeds one using estimated parameters.

## Results

**Fig 3:**
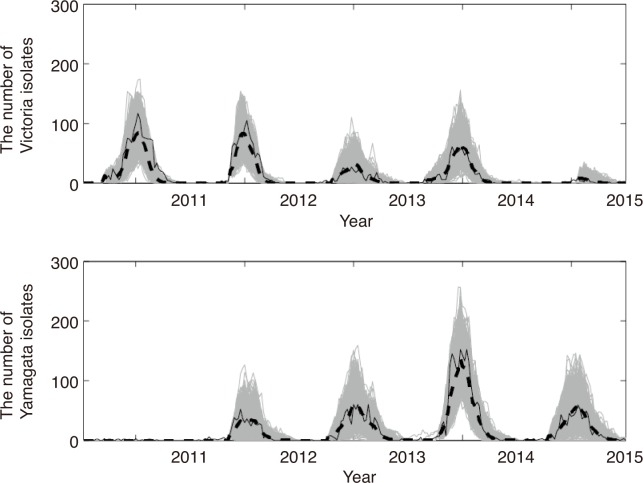
Comparison between the accepted simulations by ABC and the data from the 2010–2011 season to the 2014–2015 season. Each gray line shows the accepted simulation run by ABC. The dashed line shows the average of the accepted simulation runs by ABC. The black line shows the data of the weekly reported number of isolates. The top panel shows the isolation of Victoria lineage and the bottom panel shows the isolation of Yamagata.

**Fig 4:**
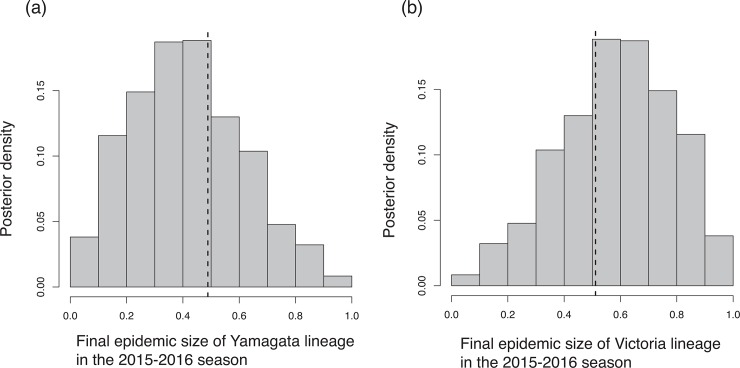
Cross-validation of our model estimation. The histogram shows the distribution of the predicted final epidemic size in the 2015–2016 season using the posteriors of parameters estimated from the weekly reported lineage-specific IBV cases from the 2010–2011 season to the 2014–2015 season. Dashed line shows the field data.

Our model captured the lineage dynamics of both Victoria and Yamagata from the 2010–2011 season to the 2014–2015 season well (Fig 3). Table 1 summarizes the estimated values of parameters; with most parameters being similar between Yamagata and Victoria, with the exception of *b* and *κ*. The amplitude of seasonal fluctuation of transmission rate for the Victoria lineage, *b*_Victoria_, is higher than that for Yamagata. *κ*_Yamagata_ is much higher than *κ*_Victoria_, the average sojourn time until the loss of immunity is 1.15 years for Victoria and 0.079 years for Yamagata. As validation of our model, we simulated the epidemics in 2015–2016 season using the estimated posterior distributions and the field data of the emergence time of Victoria and Yamagata in 2015–2016 season, and the simulated final epidemic sizes per lineage were compared to the field data (leave-one-out cross-validation). Our model can capture the final epidemic size per lineage well (Fig 4).

**Table 1:** The estimated values of the parameters in our model.

Parameters for Yamagata	*a*_Yamagata_	*b*_Yamagata_	*κ*_Yamagata_ (year^-1^)	*α*_Yamagata→Victoria_
Estimated values (95% HPD)	0.82 (0.77, 0.87)	0.77 (0.66, 0.87)	12.60 (3.96, 30.24)	0.51 (0.084, 0.92)
Parameters for Victoria	*a*_Victoria_	*b*_Victoria_	*κ*_Victoria_ (year^-1^)	*α*_Victoria→Yamagata_
Estimated values (95% HPD)	0.83 (0.74, 0.92)	1.05 (0.89, 1.20)	0.86 (0.65, 1.15)	0.62 (0.42, 0.80)

**Fig 5:**
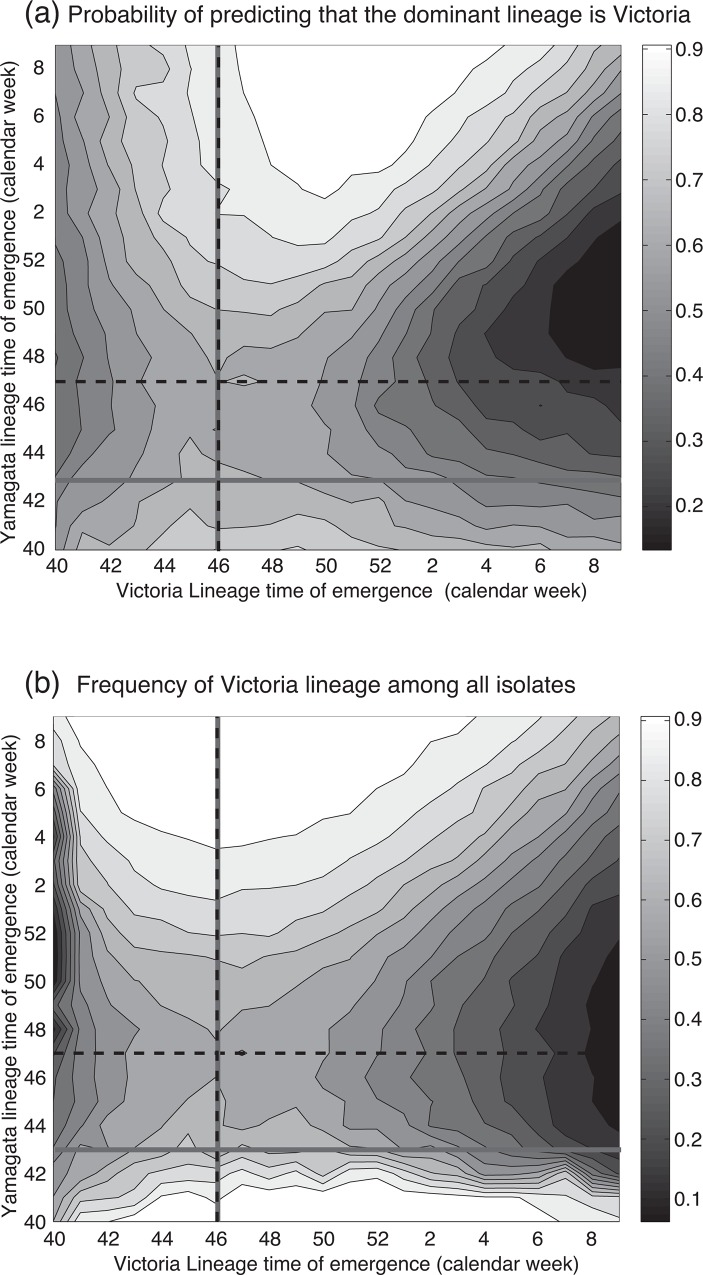
The model prediction of the epidemic in the 2015–2016 season with varied emergence timings. The straight gray lines show the actual emergence timing, and the dashed line shows the timing when the predicted *R*_0,n_ exceeds one. (a) shows the probability that the model predicts the dominant strain is the Victoria lineage. (b) shows the model prediction of the frequency of the Victoria lineage among all isolations. The actual frequency of the Victoria lineage among all isolates in the 2015–2016 season is 0.51.

Using the posterior distribution of parameters in our model we predicted the dominant lineage for the 2015–2016 season. The number of isolates for the Yamagata and Victoria lineages were close during the 2015–2016 season in Japan; 663 isolates of the Yamagata lineage and 694 isolates of the Victoria lineage were reported by 12th April 2016. Although the emergence timing plays a key role in determining the dominant lineage, at this moment we do not know the future emergence timing. The average accuracy obtained by varying the emergence timings of Yamagata and Victoria is 0.614. Fig 5A shows the sensitivity analysis of the accuracy of prediction for the dominant lineage. The accuracy was improved to 0.688 if we use the actual emergence timing.

**Fig 6:**
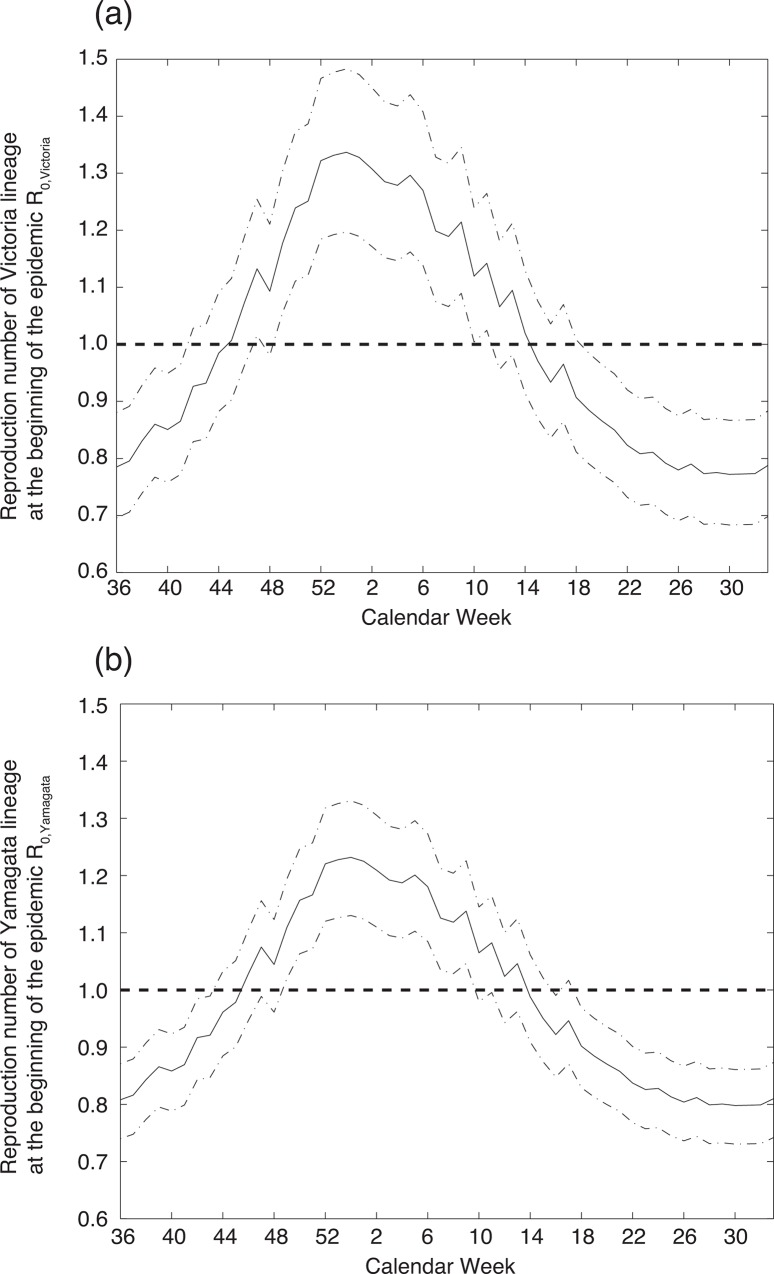
Estimated basic reproduction number at the beginning of the epidemic *R*_0,n_ in the 2015–2016 season with varied emergence timings. (a) shows *R*_0,Victoria_, and (b) shows *R*_0,Yamagata._ The solid black line shows the median of the highest posterior density (HPD) and the curved dashed lines show the lower and upper bounds of 95% HPD.

We showed that understanding the emergence timing is crucial for the prediction of the dominant lineage. We also tried to narrow down the considerable range of the emergence timing using the lineage specific basic reproduction number *R*_0,n_. Fig 6 shows *R*_0,n_ during 2015–2016 season. The calendar week when the *R*_0,n_ exceeds one is the 46^th^ week (95% highest posterior density (HPD): 43^rd^–48^th^ week) for Victoria and the 47^th^ week (95%HPD: 44^th^ -50^th^ week) for Yamagata. The actual emergence time, determined as the time when the weekly isolation number exceeds 6, is the 46^th^ and 43^rd^ week for Victoria and Yamagata, respectively. Estimated emergence timing by *R*_0,n_ can improve the accuracy of prediction, 64.6 percent of 1000 simulation runs with estimated timing shows the correct dominant strain.

Due to the similar number of isolates between Yamagata and Victoria in the 2015–2016 season, it was difficult to determine the dominant strain. We also compared our prediction to the frequency of Victoria lineage isolates among all the IBV isolates (Fig 5B). Our model can predict the frequency of lineage as well. The predicted frequency of the Victoria lineage using the observed emergence timing by *R*_0,n_ is 0.58 (95%HPD: 0.01–0.99), the predicted frequency using estimated emergence timing by *R*_0,n_ is 0.61 (95%HPD: 0.01–0.99), the average predicted frequency of Victoria among varied emergence timings is 0.64 (95%HPD: 0.12–0.97), and the observed frequency of Victoria was 0.51 from the field data.

## Discussion

In this paper we estimated the dynamics of two major IBV lineages using a parsimonious mathematical model. Although the prediction of the dominant lineage of IBV is important for vaccine strain selection, the complex lineage dynamics of IBV makes this prediction difficult. Our result suggests that the prediction of the dominant lineage of IBV may be possible if the epidemiological interference between lineages was quantified.

Our estimates of the waning rate of immunity suggest that immunity against the Yamagata lineage is shorter than immunity to Victoria. This tendency is robust even when the seasons of the data for the estimation were changed; 2010–2013, 2011–2014, 2012–2015, 2010–2014, and 2011–2015 compared to the result using 2010–2015 seasons (Table 2). There are two possible interpretations of this result: i) the antigenicity of Yamagata changes much faster than Victoria or ii) the immunity against Yamagata wanes faster than Victoria. The results of phylogenetic analysis and the antigenicity measured by hemagglutination inhibition assay suggest that the Victoria lineage is under stronger selection pressure due to host immunity, and the antigenicity of Victoria changes faster than that of Yamagata [20]. Therefore hypothesis i) is not likely to be true. On the other hand, the hypothesis ii) is not rejected by the result of phylogenetic and antigenicity analyses. If the immunity itself wanes rapidly, the lineage cannot be under selection pressure by host immunity and the change of antigenicity is not essential for the persistence of the lineage. Furthermore, the broader age-distribution of infection of Yamagata than Victoria implies frequent re-infection with the Yamagata lineage [20], supporting the possibility of rapid waning immunity against the Yamagata lineage. The clinical trial of vaccines against the Yamagata lineage showed stronger immune reaction to Yamagata than to Victoria [23], but this immunity may persist temporarily and wane rapidly. To conclude, whether the immunity against the Yamagata lineage is of long or short duration will require further study.

**Table 2:** The posterior of parameters estimated using the data in varied seasons. Each parenthesis shows the 95% highest posterior density.

Data used for the estimation	Posterior of each parameter							
	*a*_Yamagata_	*b*_Yamagata_	*κ*_Yamagata_	*α*_Yamagata→Victoria_ (year^-1^)	*a*_Victoria_	*b*_Victoria_	*κ*_Victoria_	*α*_Victoria→Yamagata_ (year^-1^)
2010–2015	0.82 (0.77, 0.87)	0.77 (0.66, 0.87)	12.60 (3.96, 30.24)	0.51 (0.08, 0.92)	0.83 (0.74, 0.92)	1.05 (0.89, 1.20)	0.86 (0.65, 1.15)	0.62 (0.42, 0.80)
2010–2014	0.77 (0.74, 0.80)	0.89 (0.84, 0.95)	14.97 (7.3, 26.65)	0.68 (0.15, 0.99)	0.91(0.8, 1.01)	1.04 (0.98, 1.09)	0.73 (0.62, 0.84)	0.57 (0.50, 0.63)
2011–2015	0.83 (0.78, 0.88)	0.84 (0.74, 0.94)	14.60 (6.57, 27.01)	0.61 (0.10, 0.99)	1.02 (0.91, 1.12)	1.20 (1.03, 1.38)	0.73 (0.62, 0.84)	0.70 (0.55, 0.82)
2010–2013	1.00 (0.92, 1.17)	0.92 (0.85, 0.99)	8.40 (1.93, 33.95)	0.38 (0.02, 0.97)	1.00 (0.92, 1.08)	0.80 (0.65, 0.95)	0.95 (0.77, 1.20)	0.44 (0.29, 0.62)
2011–2014	0.84 (0.61, 1.10)	1.14 (0.81, 1.56)	21.90 (5.11, 36.14)	0.82 (0.34, 0.99)	1.36 (1.06, 1.70)	0.98 (0.52, 1.56)	0.36 (0.27, 0.51)	0.49 (0.24, 0.79)
2012–2015	0.88 (0.79, 0.96)	0.66 (0.54, 0.79)	5.84 (2.37,19.71)	0.95 (0.69, 0.99)	0.69 (0.57, 0.84)	1.12 (0.87, 1.40)	1.35 (0.69, 3.58)	0.84 (0.45, 0.99)

Our estimates of lineage-specific reproduction numbers agree with phylodynamic analysis [20]; the average reproduction number of Victoria is larger than that of Yamagata. The seasonal fluctuation of the reproduction number of Victoria is also larger than that of Yamagata. Our estimate of the reproduction number takes into account both cross-reactivity of immunity between Victoria and Yamagata and waning immunity. If we misestimated these two factors, estimated values would be far from the estimate by phylodynamic analysis.

Our estimation suggests that the cross-immunity between Victoria and Yamagata is high enough that infections by one lineage suppress infections to the other lineage at the population level. However, cross-immunity between the two lineages cannot protect individuals from infection. This highlights the importance of the selection of the IBV vaccine lineages in the countries where trivalent influenza vaccines are used.

Prediction of strain dynamics requires long time series data of lineage specific isolates. In fact, prediction using data from only one year cannot capture the strain dynamics, and the opposite lineage of a year’s dominant lineage, as shown in the data, was selected as the dominant lineage each time (the data is not shown in this paper). Surveillance with an appropriate and consistent study design is essential for predicting the lineage dynamics for vaccine strain selection.

For simplicity, our model assumed constant antigenic evolution within each lineage. This assumption is based on the fact that no new lineage has emerged after the branching of IBV to Yamagata and Victoria, which suggests it has relatively stable evolutionary dynamics compared to IAV. Our model is sufficient for the short-term prediction of lineage dynamics, however, when we predict the emergence of new lineages and the extinction of current lineages, the evolutionary dynamics at the quasi-species level would need to be taken into account.

Even though the dominant lineage can be predicted, selection of the specific strain is still required. Especially, the antigenicity of the Victoria lineage changes rapidly, so further understanding of the evolution of the Victoria lineage is necessary. Phylodynamic studies showed that the time series change of the genetic diversity of Victoria is similar to influenza A H3N2 and H1N1, so the model must take into account evolution at the strain level [16,17,24,25].

In conclusion, we developed a parsimonious mathematical model describing the lineage dynamics of IBV. Using the weekly number of lineage specific isolates we estimated the reproduction number, the waning rate of immunity, and the strength of cross-immune reaction. Our prediction suggests that models taking into account epidemiological interference due to cross-immune reaction and the seasonality of transmission can predict the lineage dynamics of IBV for the next year.

## Supporting Information

S1 Fig The sensitivity analysis of posteriors to the host population size. x denotes the mode of the posterior distribution and error bar denotes 95% highest posterior density.
(EPS)

S2 Fig The sensitivity analysis of posteriors to the number of accepted simulation runs for the construction of the posterior distribution. x denotes the mode of the posterior distribution and error bar denotes 95% highest posterior density.
(EPS)

## References

[1] Molinari NA, Ortega-Sanchez IR, Messonnier ML, Thompson WW, Wortley PM, Weintraub E, et al (2007) The annual impact of seasonal influenza in the US: measuring disease burden and costs. Vaccine 25: 5086–5096. 10.1016/j.vaccine.2007.03.046 17544181

[2] Glezen WP (2014) Editorial commentary: Changing epidemiology of influenza B virus. Clin Infect Dis 59: 1525–1526. 10.1093/cid/ciu668 25139967

[3] Thompson WW, Shay DK, Weintraub E, Brammer L, Cox N, Anderson LJ, et al (2003) Mortality associated with influenza and respiratory syncytial virus in the United States. JAMA 289: 179–186. 12517228 10.1001/jama.289.2.179

[4] Mosterin Hopping A, Fonville JM, Russell CA, James S, Smith DJ (2016) Influenza B vaccine lineage selection-An optimized trivalent vaccine. Vaccine 34: 1617–1622. 10.1016/j.vaccine.2016.01.042 26896685 PMC4793086

[5] Shaman J, Pitzer VE, Viboud C, Grenfell BT, Lipsitch M (2010) Absolute humidity and the seasonal onset of influenza in the continental United States. PLoS Biol 8: e1000316 10.1371/journal.pbio.1000316 20186267 PMC2826374

[6] Shaman J, Kohn M (2009) Absolute humidity modulates influenza survival, transmission, and seasonality. Proc Natl Acad Sci U S A 106: 3243–3248. 10.1073/pnas.0806852106 19204283 PMC2651255

[7] Grassly NC, Fraser C (2006) Seasonal infectious disease epidemiology. Proc Biol Sci 273: 2541–2550. 10.1098/rspb.2006.3604 16959647 PMC1634916

[8] Stone L, Olinky R, Huppert A (2007) Seasonal dynamics of recurrent epidemics. Nature 446: 533–536. 10.1038/nature05638 17392785

[9] Schwartz IB, Smith HL (1983) Infinite Subharmonic Bifurcation in an Seir Epidemic Model. Journal of Mathematical Biology 18: 233–253. 6663207 10.1007/BF00276090

[10] Castillochavez C, Hethcote HW, Andreasen V, Levin SA, Liu WM (1989) Epidemiological Models with Age Structure, Proportionate Mixing, and Cross-Immunity. Journal of Mathematical Biology 27: 233–258. 2746140 10.1007/BF00275810

[11] Rohani P, Earn DJ, Finkenstadt B, Grenfell BT (1998) Population dynamic interference among childhood diseases. Proceedings of the Royal Society B-Biological Sciences 265: 2033–2041. 10.1098/rspb.1998.0537 PMC1689490 9842732

[12] Kamo M, Sasaki A (2002) The effect of cross-immunity and seasonal forcing in a multi-strain epidemic model. Physica D-Nonlinear Phenomena 165: 228–241.

[13] Gupta S, Ferguson N, Anderson R (1998) Chaos, persistence, and evolution of strain structure in antigenically diverse infectious agents. Science 280: 912–915. 9572737 10.1126/science.280.5365.912

[14] Gog JR, Grenfell BT (2002) Dynamics and selection of many-strain pathogens. Proc Natl Acad Sci U S A 99: 17209–17214. 10.1073/pnas.252512799 12481034 PMC139294

[15] Kryazhimskiy S, Dieckmann U, Levin SA, Dushoff J (2007) On state-space reduction in multi-strain pathogen models, with an application to antigenic drift in influenza A. Plos Computational Biology 3: 1513–1525. 10.1371/journal.pcbi.0030159 PMC1949840 17708677

[16] Luksza M, Lassig M (2014) A predictive fitness model for influenza. Nature 507: 57-+. 10.1038/nature13087 24572367

[17] Omori R, Sasaki A (2013) Timing of the emergence of new successful viral strains in seasonal influenza. J Theor Biol 329: 32–38. 10.1016/j.jtbi.2013.03.027 23567650

[18] Tria F, Lassig M, Peliti L, Franz S (2005) A minimal stochastic model for influenza evolution. Journal of Statistical Mechanics-Theory and Experiment.

[19] Minayev P, Ferguson N (2009) Improving the realism of deterministic multi-strain models: implications for modelling influenza A. Journal of the Royal Society Interface 6: 509–518. 10.1098/rsif.2008.0333 PMC2586098 18801714

[20] Vijaykrishna D, Holmes EC, Joseph U, Fourment M, Su YC, Halpin R, et al (2015) The contrasting phylodynamics of human influenza B viruses. Elife 4: e05055 10.7554/eLife.05055 25594904 PMC4383373

[21] Xu C, Chan KH, Tsang TK, Fang VJ, Fung RO, Ip DK, et al (2015) Comparative Epidemiology of Influenza B Yamagata- and Victoria-Lineage Viruses in Households. Am J Epidemiol 182: 705–713. 10.1093/aje/kwv110 26400854 PMC4715237

[22] Sunnaker M, Busetto AG, Numminen E, Corander J, Foll M, Dessimoz C (2013) Approximate Bayesian computation. PLoS Comput Biol 9: e1002803 10.1371/journal.pcbi.1002803 23341757 PMC3547661

[23] Skowronski DM, Hottes TS, Chong M, De Serres G, Scheifele DW, Ward BJ, et al (2011) Randomized controlled trial of dose response to influenza vaccine in children aged 6 to 23 months. Pediatrics 128: e276–289. 10.1542/peds.2010-2777 21768314

[24] Bedford T, Rambaut A, Pascual M (2012) Canalization of the evolutionary trajectory of the human influenza virus. BMC Biol 10: 38 10.1186/1741-7007-10-38 22546494 PMC3373370

[25] Ito K, Igarashi M, Miyazaki Y, Murakami T, Iida S, Kida H, et al (2011) Gnarled-trunk evolutionary model of influenza A virus hemagglutinin. PLoS One 6: e25953 10.1371/journal.pone.0025953 22028800 PMC3189952

